# Identification of transcriptome alterations in the prefrontal cortex, hippocampus, amygdala and hippocampus of suicide victims

**DOI:** 10.1038/s41598-021-98210-6

**Published:** 2021-09-22

**Authors:** Daniela Glavan, Victor Gheorman, Andrei Gresita, Dirk M. Hermann, Ion Udristoiu, Aurel Popa-Wagner

**Affiliations:** 1 Griffith University Menzies Health Institute of Queensland, Gold Coast Campus, Brisbane, QLD 4000 Australia; 2 Department of Psychiatry, University of Medicine and Pharmacy, Craiova, Romania; 3 Chair of Vascular Neurology, Dementia and Ageing Research, Department of Neurology, University Hospital Essen, University of Duisburg, Essen, Germany

**Keywords:** Molecular medicine, Diseases of the nervous system

## Abstract

Suicide is one of the leading causes of death globally for all ages, and as such presents a very serious problem for clinicians worldwide. However, the underlying neurobiological pathology remains to a large extent unknown. In order to address this gap, we have carried out a genome-wide investigation of the gene expression in the amygdala, hippocampus, prefrontal cortex and thalamus in post-mortem brain samples obtained from 20 suicide completers and 7 control subjects. By KEGG enrichment analysis indicated we identified novel clusters of downregulated pathways involved in antigen neutralization and autoimmune thyroid disease (amygdala, thalamus), decreased axonal plasticity in the hippocampus. Two upregulated pathways were involved in neuronal death in the hippocampus and olfactory transduction in the thalamus and the prefrontal cortex. Autoimmune thyroid disease pathway was downregulated only in females. Metabolic pathways involved in Notch signaling amino acid metabolism and unsaturated lipid synthesis were thalamus-specific. Suicide-associated changes in the expression of several genes and pseudogenes that point to various functional mechanisms possibly implicated in the pathology of suicide. Two genes (SNORA13 and RNU4-2) involved in RNA processing were common to all brain regions analyzed. Most of the identified gene expression changes were related to region-specific dysregulated manifestation of genetic and epigenetic mechanisms underlying neurodevelopmental disorders (SNORD114-10, SUSd1), motivation, addiction and motor disorders (CHRNA6), long-term depression (RAB3B), stress response, major depression and schizophrenia (GFAP), signal transduction at the neurovascular unit (NEXN) and inhibitory neurotransmission in spatial learning, neural plasticity (CALB2; CLIC6, ENPP1). Some of the differentially expressed genes were brain specific non-coding RNAs involved in the regulation of translation (SNORA13). One, (PARM1) is a potential oncogene and prognostic biomarker for colorectal cancer with no known function in the brain. Disturbed gene expression involved in antigen neutralization, autoimmunity, neural plasticity, stress response, signal transduction at the neurovascular unit, dysregulated nuclear RNA processing and translation and epigenetic imprinting signatures is associated with suicide and point to regulatory non-coding RNAs as potential targets of new drugs development.

According to the World’s Health Organization, nearly one million people die from suicide every year, making suicide one of the leading causes of death globally for all ages. Although many psychosocial factors such as stress, impulsive-aggressive behaviour, chronic disease and hopelessness contribute to suicidality^1–4^, associated neurobiological pathology remains to a large extent unknown. Thus, there is a need for systematic research approaches to enable better understanding of the molecular aetiology of suicide that may in turn enable new therapeutic and diagnostic approaches.

It has been proposed that mood disorders/suicide result from an inability of the brain to make appropriate adaptive responses to environmental stimuli as a result of impaired synaptic and structural plasticity. Support for this idea comes from studies demonstrating consistent changes in the expression of genes that are critical in synaptic and structural plasticity^5^.

A number of studies performed on blood cells, cerebrospinal fluid and plasma from suicidal patients have suggested the involvement of serotonergic, dopaminergic and noradrenergic systems, as well as abnormalities in hypothalamic-pituitary-adrenocortical axis^6–8^. The mechanisms underlying the pathophysiology major depression remain unclear, but recent studies demonstrate that depression and chronic stress exposure cause atrophy of neurons in cortical and limbic brain regions implicated in depression, and brain imaging studies demonstrate altered connectivity and network function in the brains of depressed patients^11^.

Non-coding RNAs, microRNAs (miRNAs) are the most studied and well characterized and have emerged as a major regulator of neural plasticity and higher brain functioning. The role of miRNAs in depression and suicidal behavior is still in its nascent stage; however, several lines of evidence, including pre-clinical and clinical, demonstrate that miRNAs may play a critical role in the development of stress-related disorders including depression and suicidal behavior^5, 10, 11^. Indeed, a study used Weighted Gene Coexpression Network Analysis (WGCNA) to identify genes associated with depression and suicide and identified 23 long non-coding RNAs (lncRNAs) are an emerging class of regulatory RNA that may be implicated in psychiatric disorders^12^.

A genome-wide SGS analysis of the PsychArray platform identified four significant PsychArray SNP variants that were linked to an increased likelihood of completed suicide^13^. Another study used available GWAS SNP data to make gene-levels associations with severe suicide attempts and found that inherited genetic variation is of importance in the developmental processes of the stress-vulnerability model in suicidality^14^. Likewise, genetic risk factors for suicide attempt severity pointed to genes involved in anaerobic energy production (LDHB), circadian clock regulation (ARNTL2), and catabolism of tyrosine (FAH)^15^. A recent study explored the association between SNP variants of the corticotropin-releasing hormone receptor 1 polymorphisms and suicidal behaviour^16^ while transcriptomics revealed important sex differences^17^.

However, in order to obtain direct information about molecular brain pathology, it is necessary to analyze postmortem brain samples of suicide victims, with the attendant detailed clinical records. Such approaches may open up new opportunities to identify and characterize genes whose expression is changed in the suicidal brain. In this study, we report the results of a transcriptomic analysis carried out on four brain regions (prefrontal cortex, amygdala, hippocampus, and thalamus) of suicide victims and controls.

## Materials and methods

The postmortem cohort consisted of 20 suicide completers. Brain tissue was obtained from The Douglas Bell Canada Brain Bank. Specific information regarding the circumstances of death and toxicology were provided by The Douglas Bell Canada Brain Bank. The control group consisted of seven cases who died of natural causes. We excluded control subjects with personal and/or familial history of psychiatric disorders and/or suicidal behaviour. All tissue was collected following informed consent obtained from the families of each deceased subject. Subjects and control demographics are given in Tables 1 and 2. All methods using human tissue were performed in accordance with guidelines established by the Griffith University Human Research Ethics Committee and after approval of the ethics committee (Ref. 2018/15).

**Table 1:** Controls demographics.

Subject ID	Age (years)	Gender	PMI	Cause of death
C34735	32	M	18	Accident at work
C18895	33	F	10	Motor vehicle crash
C34566	56	M	18	Accident at work
C33567	47	M	12	Hypothermia
C24785	47	M	9	Motor vehicle crash
C24792	42	F	11	Motor vehicle crash
C19337	39	F	27	Drowning

F, female; M, male.

**Table 2:** Demographic characteristics of subjects included in the study.

Gender	Age (years)	PMI (h)	Cause of death	Psychiatric diagnosis	Toxicological findings
M	48	32	Hypothermia	Alcohol dependence	No substances found
M	33	10	Sudden death unknown	No substances found	
F	45	15	Natural gas	MDD	Citalopram Prozac
F	26		Drug overdose	Substance dependence	Benzodiazepines Cocaine, Heroine
F	30		Drug overdose	Substance dependence Substance dependence	Benzodiazepines Cocaine
F	31	72	Drug overdose	Substance dependance	Benzodiazepines Cocaine, Heroine
F	38	50	Hanging	MDD	No substances found
F	70	38	Asphyxia	Frontal lobe atrophy	No substances found
M	48	9	Hanging	Personality disorders	Lamotrigine Olanzapine
M	30	26	Town gas	Acute paranoid psychosis	Risperidone, Olanzapine, Lorazepam
M	39	22	Hanging	MDD	Cannabis
M	62	31	Fall from the height	MDD with paranoid symptomes	No substances found
M	59	73	Asphyxia	Mental handicap	No substances found
M	27	44	Drowning	MDD Alzheimer disease	No substances found
M	50	40	Drowning	Alcohol dependence	No substances found
F	40	23	Asphyxia	Substance dependance	Olanzapine, Risperidone
M	57	31	Fall from the height	Personality disorders	Olanzapine
M	38	19	Hypothermia	Alcohol dependence	No substances found
F	40	15	Town gas	Acute paranoid psychosis	Olanzapine, Risperidone
F	42	18	Hanging	MDD	Citalopram, Mirtazapine

F, female; M, male; MDD, major depressive disorder; PMI, post-mortem.

interval.

### RNA extraction and microarray hybridisation

After the tissue was homogenized, total RNA was extracted from the hippocampus, amygdala, thalamus and prefrontal cortex of each subject using the TRIzol reagent (Invitrogen Life Technologies, Germany) as previously described^18^. Genomic DNA was removed using the RNeasy Plus kit (Qiagen, Germany). Prior to sample preprocessing, RNA integrity was assessed with the RNA 6000 nano kit using the Bioanalyzer 2100 instrument (Agilent, Germany). RNA integrity numbers ranged between 6.5 and 8.2. 200 ng of each sample were processed with the whole transcript (WT) expression kit (Ambion, Germany), i.e., subjected to RNA amplification via reverse transcription to double-stranded cDNA and subsequent in vitro transcription; this was followed by another round of reverse transcription yielding single-stranded DNA in sense orientation. Hybridisation cocktails were produced after fragmentation and biotin labeling of target DNAs following the protocol of the GeneChip WT terminal labeling kit (Affymetrix, Canada). Microarray hybridisation to GeneChip Human Transcriptome Array 2.0 (Affymetrix, Canada) was performed individually according to the manufacturer's protocol using the Fluidics Station 450 with the program FS450_0007. CEL files from scanned microarrays were produced with the expression console (Affymetrix, Canada). The hybridization experiment was repeated twice^19^.

### Microarray evaluation

Consistently high quality of microarray data was ensured by the visual inspection of scanned images for hybridization artifacts, quality metrics from the scanning and gridding procedure and correspondence analysis of microarray expression values. Normalizations were performed with the Quantiles method^20^; background correction and probe set summary were achieved with Robust Microarray Average (RMA) using the Expression Console software (build 1.3.0.187, Affymetrix, Canada). Differentially expressed genes were derived from fixed effects modelling in an empirical Bayes framework by brain region-specific comparison of suicide victims to controls; the correlation of data from different tissues of the same individual was modelled as random effect. The significance threshold for differential expression was set to p < 0.001^19^.

### KEGG pathway analysis

Gene Ontology (GO) analysis is a common and useful method for large-scale functional enrichment research. To further analyze the potential biological process, molecular function, and cellular component, upregulated and downregulated genes were analyzed for enrichment using GSEA software (www.kegg.jp/kegg/kegg1.html). GSEA calculates a normalized enrichment score (NES) that reflects any overrepresentation of predefined gene sets in suicide completers vs controls. The Database for Annotation, Visualization and Integrated Discovery (DAVID) 2007 Functional Annotation Clustering was used to search the database of the Kyoto Encyclopedia of Genes and Genomes (KEGG)^21^ in order to identify significantly over-represented pathways in the subset of differentially expressed genes. More specifically, the latter is a curated pathway database comprising biological signaling pathways that are based on current knowledge of molecular interactions involved in various cellular processes. P < 0.05 and FDR < 0.05 were considered to indicate a statistically significant difference.

### Quantitative real-time PCR

Genes that showed the highest expression changes were selected for PCR-verification. For quantitative real time PCR (qPCR), we synthesized cDNA from total RNA with the High-Capacity cDNA reverse transcription kit (Applied Biosystems, USA). The qPCR was performed in 96-well 0.1-ml thin-wall PCR plates (Applied Biosystems, USA) in the Step One Plus System (Applied Biosystems, USA). Each 20 µl reaction contained 10 µl iQ SYBR Green Master Mix (BioRad Laboratories, Canada), 2 µl gene-specific forward and reverse primer mix (Eurofins and Qiagen, Canada) and 8 µl pre-diluted cDNA. No-template controls contained nuclease-free water instead. The cycling conditions were 3 min 95 °C to activate iTaq DNA polymerase followed by 45 cycles with 30 s denaturation at 95 °C, 30 s annealing at 58 °C and 30 s elongation at 72 °C. At the end of the amplification cycles, melting curves were used to validate PCR product specificity. All samples were amplified in triplicate. Data were analyzed using the ΔΔCt method. The expression levels of genes of interest were normalized to the average of expression level of ribosomal protein 13a (RPL 13a) from the same sample. So, the relative expression for a gene of interest was defined as the ratio of expression of the gene to that of the housekeeping gene. The fold change for a gene of interest was defined as the ratio of the relative expression in the suicide cases to that in the controls as previously described^19^. Eurofinn, (Germany) provided all primers.

### Small nucleolar RNA quantification

Small nucleolar RNA, C/D Box 114 cluster (SNORD114)-10, quantification was done by RT-qPCR using the reverse transcription primer, 5'-CTCA ACTGGTGTCGTGGAGTC GGCAATTCAGTTGAGTGGACCTC-3'; forward, 5'-ACA CTCCAGCTGGGA AGA TCA A TGA TGACT-3' and the universal primer, 5'-ACTGACTGATGCAATCTCAACTGGTGTCGT GGA-3'^22^.

### miRNA quantification

miRNA quantification was done using target-specific stem-loop primers during cDNA synthesis that produces a template for real-time PCR (ThermoFischer Scientific).

### Statistical analysis of real-time PCR-data

The selected differentially expressed genes were determined by comparing ΔΔCt of expression for each gene analyzed between suicide and controls for each brain region. The ΔΔCt values of the two groups were analyzed by the two-tailed t-test. A p-value (< 0.05) was considered significant.

### Ethics approval and consent to participate

Brain tissue was obtained from The Douglas Bell Canada Brain Bank. Specific information regarding the circumstances of death and toxicology were provided by The Douglas Bell Canada Brain Bank. The control group consisted of seven cases who died of natural causes. All tissue was collected following next-of-kin consent. Subjects and control demographics are given in Tables 1 and 2. Ethical considerations on the use of human tissue were approved by the Griffith University Human Research Ethics Committee (Ref. 2018/15).

### Consent for publication

Not applicable.

## Results

### Statistical analysis of microarray data

**Figure 1:**
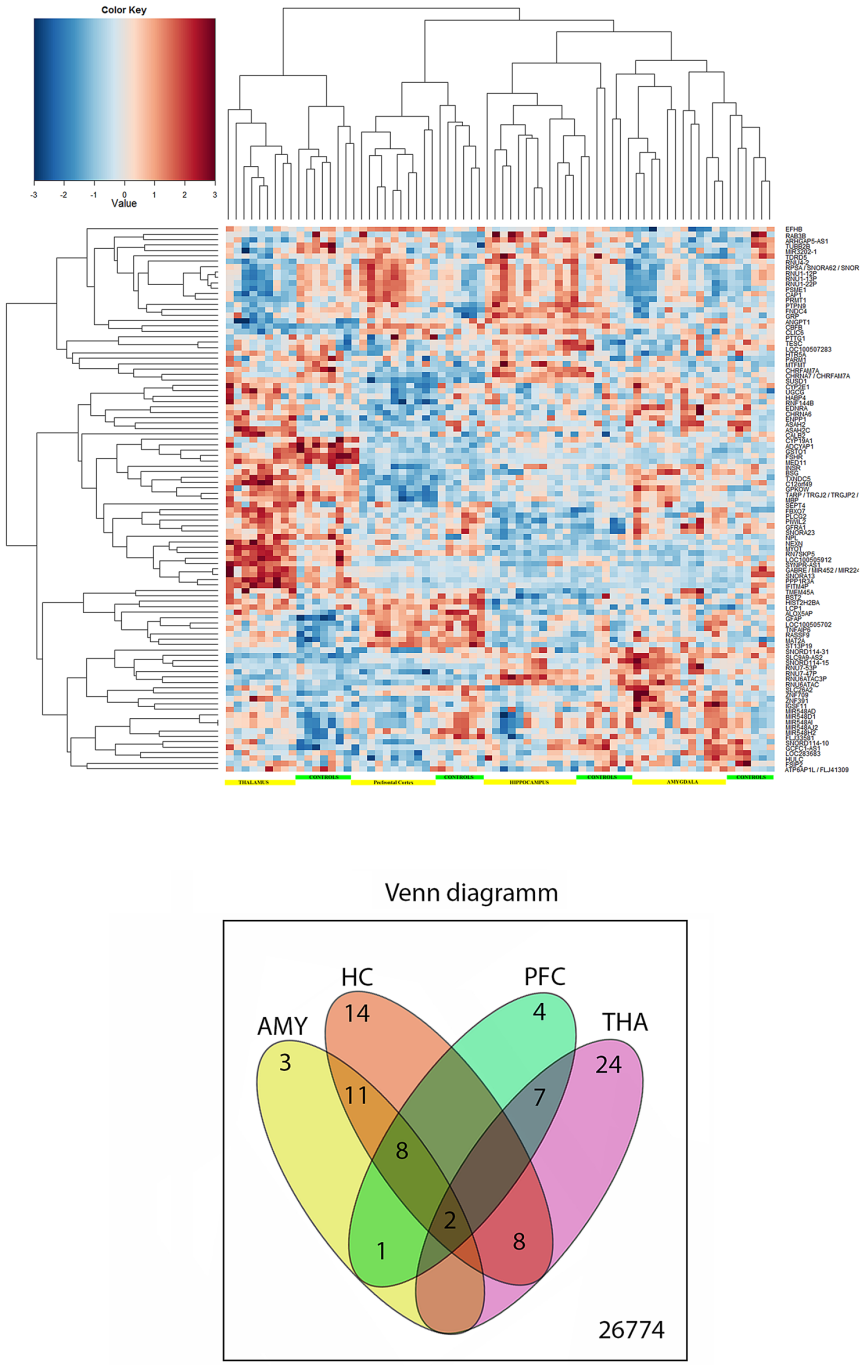
(Upper panel): Heatmap of genes differentially expressed between amygdala, hippocampus, prefrontal cortex and thalamus of suicide completers and region-specific controls (automatically generated by the Affymetrix software). Scaled expression values are shown for each group with deep blue being the lowest and dark red the highest expression level. The depicted dendrograms cluster samples (top) and genes (left) employing average agglomeration and Euclidian distance measure. (lower panel): Venn diagram showing top suicide genes that were common or specific for a brain region. AMY, amygdala; HC, hippocampus; PFC, prefrontal cortex; THA, thalamus.

Differentially expressed genes were determined by comparing suicide vs. controls for each brain region. The False Discovery Rate (FDR) of differential expression for the described comparisons was estimated with an empirical Bayes methodology employing lognormal normal data modelling which resulted in a total of 100 region-specific non-redundant probe sets. Cluster branch stability was tested with the R-package “pvclust”. Approximately unbiased p-values calculated by multiscale bootstrap resampling are shown at cluster branches. Expression values thereof were subjected to agglomerative hierarchical clustering and results were displayed as a heat map (Fig. 1). Variants for the top 100 genes were thresholded at p < 0.05 uncorrected (with either dominant or allelic model), while whole-exome results were thresholded at p < 0.0001 uncorrected. Next, the differentially expressed genes were filtered by average fold change (FC ≥|1.3|) and a FDR adjusted p-value (< 0.05) of significance was selected. Finally, the results were adjusted for sex, age, RIN, and medication status, and resulted in the identification of 100 differentially expressed genes (Suppl. Table 1).

### Gene set enrichment analysis

Pathway crosstalk analysis revealed two large clustered modules, both of which had important implications to MDD (Table 3). The first cluster included 7 pathways, and it was dominated by downregulated pathways involved in the immune response in the amygdala and thalamus. One pathway, involved in neuronal death was upregulated in the hippocampus. The second cluster was dominated by downregulated metabolic processes, most of them in the thalamus. Two pathways involved in brain plasticity were downregulated in the hippocampus, KEGG_AXON_GUIDANCE and KEGG_RNA. Of note, one upregulated pathway was KEGG_OLFACTORY_TRANSDUCTION both in thalamus and prefrontal cortex. Other upregulated pathways in the thalamus were KEGG_LONG_TERM POTENTIATION, KEGG_OXIDATIVE PHOSPHORYLATION, KEGG_CARDIAC_MUSCLE CONTRACTION and KEGG_PARKINSONS_DISEASE.

**Table 3:**
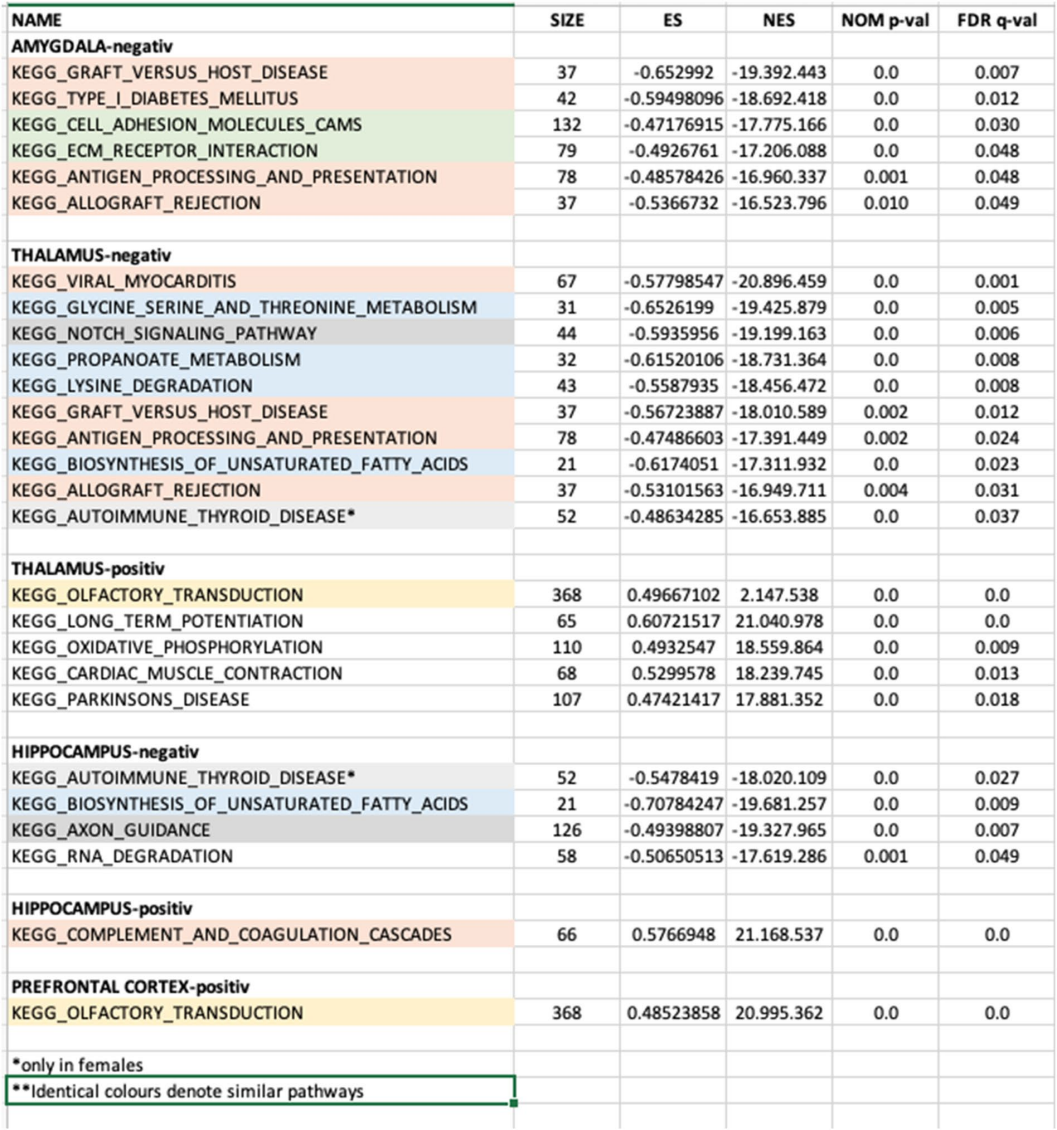
KEGG enrichment analysis of biologically relevant transcriptional programs in suicide completers (www.kegg.jp/kegg/kegg1.html).

### Differentially expressed genes according to brain location: the Venn diagram

Statistical analysis of microarray data showed that of 26774 gene sets, 44 top genes were differentially regulated between controls and suicide samples in the four brain regions analyzed (Table 4). We found that two upregulated genes (SNORA13, RNU4-2) were common to all brain regions. Eight upregulated genes (CYP2E1, ALOX5AP, LCP1, SNORA13, SNORA62, GPKOW, PRMT1) and one downregulated gene (FSIP2) were common to 3 brain regions. Top suicide genes are shown in Supplementary Table 1. Not surprisingly, eleven genes were common between amygdala and hippocampus. Six genes (GSTO1, NPL, PARM1, RNU-22P, ATP6AP1L, ENDRA and SLCA9-AS2) were common either to HC and THA or PFC and THA. One gene (TUBB2B) was common to AMY and PFC. Remarkably, there was no single gene common both to HC and PFC. Genes that were specific for a brain region are shown in Table 3. Most the region-specific genes were identified in the thalamus, 24. Finally, 15 genes that were representative for each KGG pathway (see below), were verified by RT-PCR (Table 5).

**Table 4:** RT-qPCR-based validation of brain region-specific transcripts.

Amygdala				
Affy.ID	logFC	P-Value	Gene symbol	Gene name
TC18000211.hg.1	− 0.285	0.000	GRP	Gastrin-releasing peptide
TC17001588.hg.1	− 0.481	0.000	GFAP	Glial fibrillary acidic protein
TC14001019.hg.1	0.322	0.001	ARHGAP5-AS1	ARHGAP5 antisense RNA 1 (non-protein coding)
**Hippocampus**				
TC62000125.hg.1	0.408	0.001	IFITM4P	Interferon induced transmembrane protein 4 pseudogene
TC02001689.hg.1	0.246	0.000	FNDC4	Fibronectin type III domain containing 4
TC17000602.hg.1	− 0.306	0.021	RNU6ATAC3P	RNA, U6atac small nuclear 3, pseudogene
TC06004109.hg.1	0.262	0.000	TXNDC5	Thioredoxin domain containing 5 (endoplasmic reticulum)
TC05000896.hg.1	0.262	0.000	PTTG1	Pituitary tumor-transforming 1
TC06000120.hg.1	0.239	0.001	RNF144B	Ring finger protein 144B
TC19002684.hg.1	− 0.218	0.000	ZNF709	Zinc finger protein 709
TC01003789.hg.1	− 0.254	0.000	ST13P19	Suppression of tumorigenicity 13 (colon carcinoma)
TC15002771.hg.1	− 0.257	0.001	LOC283683	Uncharacterized LOC283683
TC12001786.hg.1	− 0.353	0.000	RASSF9	Ras association (RalGDS/AF-6) domain family
TC21000138.hg.1	0.782	0.001	CLIC6	Chloride intracellular channel 6
TC17000048.hg.1	0.342	0.000	MED11	Mediator complex subunit 11
TC06000241.hg.1	− 0.315	0.000	ZNF391	Zinc finger protein 391
TC19001111.hg.1	0.190	0.001	INSR	Insulin receptor
**Prefrontal cortex**				
TC18000585.hg.1	0.304	0.001	MBP	Myelin basic protein
TC11000172.hg.1	0.501	0.001	SNORA23	Small nucleolar RNA, H/ACA box 23
TC16000528.hg.1	0.312	0.001	CBFB	Core-binding factor, beta subunit
TC05000825.hg.1	− 0.263	0.000	SLC26A2	Solute carrier family 26 (sulfate transporter), member 2
**Thalamus**				
TC08001182.hg.1	0.924	2.2756E−08	CHRNA6	Cholinergic receptor, nicotinic, alpha 6 (neuronal)
TC06000985.hg.1	0.351	2.1088E−07	ENPP1	Ectonucleotide pyrophosphatase/phosphodiesterase 1
TC09001490.hg.1	0.469	1.4837E−06	SUSD1	Sushi domain containing 1
TC10001287.hg.1	0.299	1.9713E−05	ASAH2	N-acylsphingosine amidohydrolase (ceramidase 2)
TC10000781.hg.1	0.272	2.217E−05	GSTO1	Glutathione S-transferase omega 1
TC01001554.hg.1	0.254	2.5522E−05	TDRD5	Tudor domain containing 5
TC01002654.hg.1	1.090	3.399E−05	RAB3B	RAB3B, member RAS oncogene family
TC08000169.hg.1	0.228	3.7892E−05	PIWIL2	piwi-like 2 (Drosophila)
TC16000589.hg.1	1.121	8.6087E−05	CALB2	Calbindin 2
TC02001831.hg.1	0.311	0.000	FSHR	Follicle stimulating hormone receptor
TC10001246.hg.1	0.358	0.000	ASAH2C	N-acylsphingosine amidohydrolase (non-lysosomal ceramidase) 2C
TC13000180.hg.1	− 0.494	0.000	RN7SKP5	RNA, 7SK small nuclear pseudogene 5
TC16000643.hg.1	0.347	0.000	PLCG2	Phospholipase C, gamma 2 (phosphatidylinositol-specific)
TC01000789.hg.1	− 0.661	0.000	NEXN	Nexilin (F actin binding protein)
TC03001679.hg.1	− 0.245	0.000	IGSF11	Immunoglobulin superfamily, member 11
TC03001519.hg.1	0.231	0.000	SYNPR-AS1	SYNPR antisense RNA 1 (non-protein coding)
TC18000007.hg.1	0.403	0.000	ADCYAP1	Adenylate cyclase activating polypeptide 1 (pituitary)
TC0X001491.hg.1	0.323	0.000	GABRE / MIR452 / MIR224	GABA) A receptor, epsilon / microRNA 452 / microRNA 224
TC15001362.hg.1	0.264	0.001	CYP19A1	Cytochrome P450, family 19, subfamily A, polypeptide 1
TC05000689.hg.1	− 0.312	0.001	MYOT	Myotilin
TC21000120.hg.1	− 0.296	0.001	GCFC1-AS1	GCFC1 antisense RNA 1 (non-protein coding)
TC09000560.hg.1	0.297	0.001	UGCG	UDP-glucose ceramide glucosyltransferase
TC09000480.hg.1	0.180	0.001	HABP4	Hyaluronan binding protein 4
TC02000511.hg.1	− 0.333	0.001	MAT2A	Methionine adenosyltransferase II, alpha

**Table 5:** Differentially expressed genes according to brain location.

Gene	Function	Amygdala		HC		Prefrontal cortex		Thalamus	
		*fc*	P-value	*fc*	P-value	*fc*	P-value	*fc*	P-value
CALB2*	Neurogenesis; neuroplasticity							2.17	0.01
CLIC6*	Inhibitory neurotransmission; spatial learning and neuroplasticity			1.72	0.01				
CX3CR1*	Neuroinflammation	2.96	0.001	2.34	0.01			3.78	0.001
CHRNA6*	Motivation, addiction and motor disorders							1.9	0.01
C3*	Microglia-dependent synaptic plasticity	2.24	0.001	2.34	0.01			3.78	0.001
ENPP1*	Maintenance of stem cell phenotype							1.75	0.001
GFAP*	Neuroinflammation; major depression (MDD) and schizophrenia	1.92	0.001						
MIR548H2**	microRNA, regulation of stability and translation of mRNA			− 2.73	0.001				
NEXN*	Neuroplasticity through cytoskeleton, signal transduction at the neurovascular unit							1.84	0.01
PARM1*	Potential oncogene; prognostic biomarker for colorectal cancer			1.95	0.001			1.71	0.001
RAB3B*	Long-term depression; short-term plasticity; normal reversal learning							2.13	0.01
RNU4-2*	RNA, U4 small nuclear 2	1.93	0.01	2.15	0.01	1.85	0.01	2.30	0.01
RNU6ATAC*	Cellular stress; aggressive neuroblastoma			− 1.77	0.01				
RNU7-53P*	Diabetes and related traits	− 1.82	0.01						
RNU7-47P*	Pre-mRNA intron splicing regulation							− 1.70	0.01
SNORD114-10*	Neurodevelopmental disorders			− 2.34	0.001				
SNORA13*	rRNA modification	2.24	0.001	2.3	0.001	1..85	0.001	2.25	0.001
SUSD1*	Epigenetic signature							2.42	0.001
TRHR*	Serotoninergic neurotransmission			− 3.48	0.001				

*fc*, fold change; *Verified by RT-qPCR; **Verified by Stem-loop RT-qPCR.

HC, hippocampus.

### Functional analysis of differentially expressed genes

Genes that were new for suicide or showed a region-specific expression were further verified by RT-PCR or Stem-loop RT-PCR.

The CALB2 (calbindin 2) gene encodes an intracellular calcium-binding protein belonging to the troponin C superfamily. This protein plays a role in diverse cellular functions, including message targeting and intracellular calcium buffering. It also functions as a modulator of neuronal excitability and protects against apoptosis^23^. Moreover, calbindin has been involved in the neuroplasticity underlying emotional memory^24^.

RAB3B encodes a Ras-related protein Rab-3B, which is involved in the regulation of pituitary hormone secretion through its role in the process of exocytosis by the pituitary cells^25^. In addition, the Ras-related protein, Rab-3B may be involved in polarized transport of apical, basolateral, and tight junctional membrane proteins to the plasma membrane the regulation of synaptic plasticity^26, 27^.

The PARM1 gene encodes a prostate androgen-regulated mucin-like protein 1. PARM1 has first been described as a transcript specifically upregulated in the rat ventral prostate after castration^28^ and, since it is involved in the cell proliferation enhancement, it has been suggested as a potential oncogene and prognostic biomarker for colorectal cancer^29, 30^. However, no specific function of this transcript in the brain has been described to date.

The product of the GFAP gene is one of the major intermediate filament proteins of mature astrocytes and a specific marker for astrocytes known to be involved in the pathogenesis of major depression (MDD) and schizophrenia^31–34^.

CHRNA6 encodes one of the alpha subunits of the nicotinic cholinergic (nACh) receptor and can exert a wide range of influences through Ca2 + signals, from changes in synaptic plasticity underlying cognition, memory to events involved in neural development and neuroprotection^35^. However, their specific function in the thalamus is yet unknown.

NEXN (nexilin) encodes a filamentous actin-binding protein that may function in cell adhesion and migration. Nexilin could regulate the formations of stress fibers and focal adhesions, both playing an important role in the functioning of the cytoskeleton and intracellular signaling^36^.

Genome-wide gene expression analysis and nucleoside and nucleotide profiling revealed that knockdown of ENPP1 affects purine and pyrimidine metabolism. The ecto-nucleotidase ENPP1 (ectonucleotide pyrophosphatase/phosphodiesterase 1) was found to be highly expressed in GSCs compared with normal brain and neural stem cells. Knockdown of ENPP1 in cultured GSCs resulted in an overall downregulation of stem cell-associated genes, induction of differentiation into astrocytic cell lineage and increased cell death^37^.

SUSD1 is a DNA methylation-driven gene (MDGs) required for the development of an epigenetic signature and has been involved in venous thromboembolism^38^.

CX3CR1 is part of the fractalkine signaling which comprises the chemokine CX3CL1, mainly expressed by neurons. We found that a threefold increase in CX3CR1 transcripts in amygdala, hippocampus and thalamus suggesting a widespread neuroinflammation in the limbic system.

The raphe nuclei of the serotoninergic system play a role in many psychiatric disorders. Within the raphe nuclei, subgroups of serotonergic neurons have co-transmitters or peptides, including thyrotropin-releasing hormone (TRHR) which showed a large decrease (−3, fivefold) in the hippocampus only.

CLIC6 encodes a chloride intracellular channel co-localized with the D2-like dopamine receptors^39^. D2 receptors, localized in the mossy cells in the hilar region of the hippocampus^40^, have been involved in the process of spatial learning^41^.

MIR548H2 encodes a microRNA (mir-548), which affect stability and translation of mRNA^42^. Functional enrichment analysis showed that the mir-548 gene family play important roles in multiple biological processes and among others were involved in regulation of the actin cytoskeleton, MAPK signaling pathway and glioma progression^43^.

SNORA13 and RNU4-2 genes were found to be up-regulated in all analyzed regions with an expression fold change ranging from 1.86 in thalamus to 2.5 in the prefrontal cortex. SNORA has a nucleolar localization and has been involved in the conversion of one or more primary RNA transcripts into one or more mature RNA molecules (GO:0006394). Similarly, RNU4-2 (RNA, U4 Small Nuclear 2) is an RNA Gene, and is affiliated with the snRNA (U4 spliceosomal RNA) class related to mRNA splicing (GO:0005687) along with UU4/U6xU5 tri-snRNP complex (GO:00046540) involved in RNA transport and mRNA processing (spliceosome).

SNORD114-10, belong to the family of small nucleolar RNAs (Small Nucleolar RNA, C/D Box 114-10) and are expressed specifically in the brain. They are located in the imprinted human 14q32 locus^44^ and may play a role in the dosage compensation phenomenon and epigenetic imprinting process^45^.

RNU6ATAC [U6atac Small Nuclear (U12-Dependent Splicing; U6ATAC), form a base-paired di-snRNP complex that is essential for pre-mRNA splicing of the major class of metazoan nuclear introns and has been implicated in the cellular response to stress^46, 47^.

Two genes, RNU7-47P and RNU7-53P encode small nuclear RNA pseudogenes. The function of these pseudogenes is presently unknown. The related gene RNU4-2, which encodes a member of the small nuclear U4 RNA family, is involved in pre-mRNA intron splicing regulation^46^ and plays a role in the transcriptional flexibility of the cell.

## Discussion

The identification of suicide-related genes remains a significant challenge in molecular psychiatry. In this study, we report novel clusters of downregulated pathways involved in antigen neutralization and autoimmune thyroid disease (amygdala and thalamus) and decreased axonal plasticity in the hippocampus. Two upregulated pathways were involved in neuronal death in the hippocampus and olfactory transduction in the thalamus and the prefrontal cortex. Suicide-associated changes in the expression of several genes and pseudogenes that point to various functional mechanisms possibly implicated in the pathology of suicide. Two genes (SNORA13 and RNU4-2) involved in RNA processing were common to all brain regions analyzed. Most of the identified gene expression changes are related to region-specific dysregulated manifestation of genetic and epigenetic mechanisms underlying neurodevelopmental disorders (SNORD114-10, SUSd1), motivation, addiction and motor disorders (CHRNA6), long-term depression (RAB3B), stress response, major depression and schizophrenia (GFAP), signal transduction at the neurovascular unit (NEXN) and inhibitory neurotransmission in spatial learning, neural plasticity (CALB2; CLIC6, ENPP1). Some of the differentially expressed genes were brain specific non-coding RNAs involved in the regulation of translation (SNORA13). One, (PARM1) is a potential oncogene and prognostic biomarker for colorectal cancer with no known function in the brain. In the following we discuss these genes and the underlying pathways potentially involved in suicidality.

Recent work indicates alterations in local shape volumes of subcortical components of the cortico-striatolimbic systems that subserve emotion and impulse regulation and include prefrontal, cingulate, and insula cortices, amygdala, hippocampus, thalamus, and striatum regions^49^. Therefore, it was not surprising that the affective processing regions amygdala and thalamus shared most of the downregulated pathways involved in antigen neutralization and autoimmune thyroid disease. Similar changes in the immune system and thyroid hormones synthesis have been reported for MDD by using network- and pathway-based methods of published literature. However, it was not specified what material has been analyzed^50^. Quite recently, higher serum levels of thyroid stimulating hormone (TSH) were also correlated with suicide attempts in MDD patients with comorbid anxiety symptoms^51^.

Under-expressed genes coding for components of innate immunity and inflammatory cytokines associated with suicide-completion have been reported in the Postmorten Anterior Insula^52^.

Amygdala-specific pathways are involved in cell adhesion. Indeed, alterations in cell adhesion molecules have been reported for the brains depressed, bipolar, and schizophrenic subjects^53–55^.

Thalamus-specific downregulated pathways included several metabolic pathways dominated by several amino acids (glycine, serine, threonine, lysine) metabolism and notch signaling pathway. Indeed, abnormal levels of several amino acids have been recently identified in the peripheral blood of MDD patients^56^. Notch signaling is important in regulating neural cell proliferation, differentiation, and neural cellular growth, and is considered as a contributor in adaptive and innate immune responses and alterations in Notch signaling have been reported in mood disorders^57^.

The thalamus and hippocampus shared two downregulated pathways involved in biosynthesis of unsaturated fatty acids and thyroid autoimmune disease. Noteworthy, the healthy brain environment is enriched in long-chain polyunsaturated fatty acids maintained by endothelial cells and astrocytes^58^. KEGG pathway, autoimmune thyroid disease was downregulated only in brain samples from women. Thyroid autoimmunity has been found in association with major depression in several studies^59^. Thus, TSH receptor antibodies might be a biomarker of immune dysfunction in depression in women^60, 61^. In the adult brain, THR-*α* is most highly expressed and constitutes 70–80% of THR distribution^62^. Administration of TRH at a dose of 500 μg parenterally to unipolar depressed women led to a significant improvement in depression ratings^63, 64^.

Functional connectivity in orbitofrontal-thalamic functional connectivity has been reported in patients with MDD^65^. In addition, s*uicide* action was associated with abnormal activity in the medial PFC^66, 67^. We found that thalamus and the prefrontal cortex shared one upregulated pathway involved in olfactory transduction. Indeed, olfactory transduction pathway has been associated with suicide ideation^68, 69^.

Other upregulated pathways in the thalamus are related to long-term potentiation, Parkinson’s disease, cellular energy, including cardiac muscle contraction. Similar changes in cardiac muscle contraction have been reported for MDD by using network- and pathway-based methods of published literature. However, it was not specified what material has been analyzed^50^. Abnormal activity of genes that are required for long-term potentiation could be related to an increased microglia activity in the thalamus^70^.

Thalamus and hippocampus shared only one upregulated pathway possibly involved in microglia-mediated synaptic plasticity. Recent work has revealed that microglial cells are critical mediators of synaptic sculpting and reorganization via C3-dependent phagocytosis of synapses and neurotrophic factors for synapse function and development^71, 72^. In addition, two other pathways involved in hippocampal plasticity axonal guidance and RNA degradation, were downregulated specifically in the hippocampus.

Studies have suggested that exaggerated neuroinflammation contributes to the pathogenesis of depressive disorder^73^. A major player in neuroinflammation is activated microglia which express CX3CL1 fractalkine receptor CX3CR1. Of note, CX3CL1 is expressed mainly by neurons suggesting an engulfment of non-healthy neurons by activated microglia^74^. Indeed, three brain regions, amygdala, hippocampus showed consistent upregulation of CX3CR1 transcripts suggesting an active role of microglia in suicide ideation.

### Changes in gene expression associated with neuroplasticity

The disturbance of neural plasticity underpinning adult neurogenesis, changes in synapse structure such as debranching apical dendrites or spine loss has been suggested as one of the neurobiological mechanisms underlying psychiatric disorders^75^. Moreover, it has been proposed that suicide itself might be connected to neurobiological changes distinct from those observed in different psychiatric disorders, including pathological neuroplasticity^76^. For example, CALB2 has been identified as a key regulator of the early development of the zebrafish^77^ and has been involved in hippocampal, olfactory bulb and thalamus neurogenesis^78, 79^. According to this theory, the individuals who commit suicide are neurobiologically predisposed to hopelessness and are deficient in finding alternative life-extending solutions when facing adverse events because of insufficient adaptive abilities of their brain.

CLIC6 may modulate learning and neural plasticity through an as yet unknown interaction with the D2 receptors. In the brain, CLIC6 is mostly expressed in the hippocampus and has been implicated in psychogenic stress and dopamine receptor-mediated signaling in chronic stress which can contribute to brain pathology^80^.

RAB3B immunoreactivity has been reported in human pituitary adenoma^81^. In mouse models, Rab3B is required for long-term depression of hippocampal inhibitory synapses, short-term plasticity and for normal reversal learning^82^.

### Changes in gene expression associated with brain and behavioral disorders

We identified two genes, NEXN and GFAP, involved in the functioning of the neurovascular unit those expression was changed in suicide samples. They encode elements of the cytoskeleton being involved in the functioning and maintenance of blood vessels but also in neuroinflammation (GFAP). Indeed, glial dysfunction may be a major pathophysiological feature of mood disorders^83^. An activation of the immunological system has been described previously in suicidal subjects, with changes in cytokine and chemokine levels, reported in blood, plasma or cerebrospinal fluid (CSF) of suicide attempters or patients with suicidal ideation^84^. Post-mortem analyses of the prefrontal cortex of suicide victims seem to confirm the involvement of inflammation in suicide^85^.

Nexilin is a pivotal component of the junctional membrane complex is necessary for maintaining the transverse-axial tubular system in adult cardiomyocytes and mutations in NEXN cause cardiomyopathy in patients and animal models^86, 87^. In the brain, NEXN has been localized at cell–matrix adherens junctions and is a target for FOXP1, a gene involved in autistic behavior, language impairment, and poor intellectual performance^88^.

The nicotinic cholinergic (nACh) receptor been proposed as a candidate for bipolar disorder, nicotine, cocaine and alcohol dependence^89^. A possible region-specific role of α6-subunit-containing nicotinic receptors in the attention and motivation as well as in the development of addiction and motor disorders in the midbrain has been described^90^.

Cholinergic signaling is involved in regulating the transmission of the sensory stimuli to the associative cortex and the perception of its behavioural relevance Furthermore, it modulates the sleep–wake rhythm through regulating the thalamocortical arousal. Thus, dysregulation of this system may result in the disruption of sleep–wake rhythms, behavioural demotivation and apathy, as well as indifference to the normally relevant stimuli^91^. All these behavioural changes can often be observed in suicidal patients. In this context, the disturbed perception of the sensory stimuli could cause the indifference to the surroundings and inability to properly interpret as well as react to the events, which may in turn lead to the inability to cope with stress or to adjust to the environmental changes, resulting in suicidal thoughts and actions. Since nACh receptors convey the excitatory signals in the thalamus^92^, we propose that the up-regulation of the α6-subunit of cholinergic receptors may compensate for decreased excitatory cholinergic input in this brain region.

### Regulatory non-coding RNAs as potential targets of new drugs development

Some novel genes were found to be differentially expressed across the examined brain regions included those encoding different small nucleolar RNAs (SNORA13, SNORD114-10), micro RNAs (mir-548), and small nuclear RNAs (RNU6ATAC, RNU7-47P, RNU7-53P). Thus, SNORA13, the box H/ACA snoRNAs, are organizers of nucleolar RNA is characterized by characterized by hairpins complementary to ribosomal RNA separated by the H-box region and terminated with an ACA motif that guides for methylation, pseudo-uridylation and acetylation of rRNA^93^.

In vitro, SNORA13 induce resistance to Dox in human osteosarcoma, by modulating the expression of genes involved in DNA damaging sensing, DNA repair, ribosome biogenesis, and proliferation^94^. Its function in the brain is not yet known.

Small Nucleolar RNA, C/D Box 114-10 (SNORD114-10) has been implicated in Prader-Willi and Angelman syndromes which are neurodevelopmental disorders associated with mental retardation caused by genetic and epigenetic mechanisms involving tandemly-repeated C/D snoRNA genes at the imprinted human 14q32 domain^95, 96^. Importantly, in a mouse model of learning through the use of contextual fear conditioning, the hippocampal expression of certain snoRNAs was linked to the process of establishing an association between stimuli and their negative consequences^93^. The authors concluded snoRNAs can play an important role in immediate neurobiological events underlying higher brain functions. The expression of different snoRNAs has been also found to be developmentally regulated in neurons^97^ and to be modulated by metabolic stress^98^.

The spliceosome catalyzes the excision of introns from pre-mRNA in two steps, branching and exon ligation, and is assembled from five small nuclear ribonucleoprotein particles (snRNPs; U1, U2, U4, U5, U6) and numerous non-snRNP factors^99^.

Non-coding RNAs play a crucial role in the regulation of gene expression and translation processes and ensure a flexible response of a cell to different stimuli. Thus, mir-548 downregulation is required for cell proliferation has been associated with increased glioblastoma cell motility and could be a predictor for progression of neuroblastomas^100, 101^. High levels of Mir-548 have been found patients with early-onset Myasthenia Gravis^102^.

Many putative target genes for miRNA play a role in neuronal development^103^, synaptogenesis and other neuronal processes^104^ and have been implicated in a number of psychiatric disorders, including schizophrenia, MDD, bipolar disorder and dementias^105, 106^.

One gene found to be differentially expressed in our transcriptomic analysis, namely RNU6ATAC, has been associated with aggressive neuroblastoma and cellular response to stress^78, 107^. Since suicidality is often described as a pathological self-destructive reaction, especially in response to severe stress, we hypothesize that RNU6ATAC could be part of a pathological stress response that may precede suicidality. Finally, one study implicated RNU-53P in diabetes and related traits^108^. Thus, our finding of deregulated gene expression in the diabetes I pathway in the amygdala confirms this report.

In the light of our findings involving altered levels of non-coding RNAs in suicide victims, an intriguing possibility emerges that the differently expressed miRNAs, snoRNAs and snRNAs might potentially be used as potential targets of new drug development with which to prevent suicidality.

*Study limitations*. There are a number of important caveats that apply to the current study. Our analysis was constrained by the intrinsic limitations associated with the study of postmortem brain tissue and a small and heterogenous number of subjects. This may limit statistical power to detect lower magnitude changes, and may also increase the risk of type I error. The nature of suicide completion is such that differences in demographics, clinical histories and mode of death will result in heterogeneity in the study sample. It may be important indeed to embrace such variance as being inherent in the study of clinically-relevant populations. According to the Venn diagram, there were 3 genes specific for amygdala and 4 genes were specific for thalamus of suicide completers. The low number of genes that were changed in these two regions of suicide completers could have been due to (i) small sample size; (ii) uneven number of control and suicide samples; (iii) long PMI of some samples. However, we believe we have taken a conservative statistical approach in the light of these concerns, but propose that the promising findings from the current study should be elaborated on in future larger studies. However, given the importance of analyzing post mortem brain samples in the pressing area of suicide research, we believe that the current report indicates interesting and important new avenues of research that may in future inform clinical practice regarding suicidality.

## Conclusions

KEGG enrichment analysis indicated we report novel clusters of downregulated pathways involved in antigen neutralization and autoimmune thyroid disease (amygdala and thalamus) and decreased axonal plasticity in the hippocampus. Two upregulated pathways were involved in neuronal death in the hippocampus and olfactory transduction in the thalamus and the prefrontal cortex. One pathway (autoimmune thyroid disease) that was significantly enriched in females but not in males. We also uncovered the potential detrimental role of microglia in neural plasticity. Disturbed gene expression involved in spatial learning, stress response, major depression and schizophrenia, signal transduction at the neurovascular unit and dysregulated translation may constitute molecular pathological changes potentially associated with suicide and point to regulatory non-coding RNAs as potential suicide-risk mitigating agents. Further studies on a larger and more homogenous groups of samples are needed to confirm these results.

## Supplementary Information

Supplementary Information.

Supplementary Information.

Supplementary Information.

Supplementary Information.

## Data Availability

The datasets analyzed during the current study are not publicly available but are available from the corresponding author on reasonable request.

## Abbreviations

SNP: Single-nucleotide polymorphism
WGCNA: Weighted gene coexpression network analysis
lncRNAs: Long non-coding RNAs
LDHB: Lactate dehydrogenase B
ARNTL2: Aryl hydrocarbon receptor nuclear translocator like 2
FAH: Fumarylacetoacetate hydrolase
FDR: False discovery rate
FC: Fold change
PCR: Polymerase chain reaction
qPCR: Quantitative real time polymerase chain reaction
CALB2: Calbindin 2
CHRNA6: Cholinergic receptor nicotinic alpha 6 subunit
CLIC6: Chloride intracellular channel 6
GFAP: Glial fibrillary acidic protein
MIR548H2: MicroRNA 548 h-2
NEXN: Nexilin
PARM1: Prostate androgen-regulated mucin-like protein 1
RAB3B: Ras-related protein Rab-3B
RNU6ATAC: U6atac Small Nuclear (U12-Dependent Splicing; U6ATAC)
RNU7-47P; RNU7-53P: Small nuclear RNA pseudogenes
SNORA13: Small nucleolar RNA: H/ACA box 13
SNORD114-10: Small nucleolar RNA: C/D box 114-10
p75NTR: P75 neurotrophin receptor
MAPK: Mitogen-activated protein kinase
LDLR: Low-density lipoprotein receptor
MDD: Major depressive disorder
CSF: Cerebrospinal fluid
