# Supplementary material for: Identification of transcriptome alterations in the prefrontal cortex, hippocampus, amygdala and hippocampus of suicide victims
Source: Sci Rep. 2021 Sep 22;11:18853. doi: 10.1038/s41598-021-98210-6 (PMC8458545; doi:10.1038/s41598-021-98210-6)
Supplement: Supplementary file 4 — Supplementary Information. [file 41598_2021_98210_MOESM4_ESM.docx]

| **Table 4.** RT-qPCR-based validation of brain region-specific transcripts | | | | | | | | | |
| --- | --- | --- | --- | --- | --- | --- | --- | --- | --- |
| **GENE** | **FUNCTION** | **AMYGDALA** | | **HC** | | **PREFRONTAL CORTEX** | | **THALAMUS** | |
|  |  | ***fc*** | **P-value** | ***fc*** | **P-value** | ***fc*** | **P-value** | ***fc*** | **P-value** |
| CALB2* | neurogenesis; neuroplasticity |  |  |  |  |  |  | 2.17 | 0.01 |
| CLIC6* | inhibitory neurotransmission; spatial learning and neuroplasticity |  |  | 1.72 | 0.01 |  |  |  |  |
| CX3CR1* | neuroinflammation | 2.96 | 0.001 | 2.34 | 0.01 |  |  | 3.78 | 0.001 |
| CHRNA6* | motivation, addiction and motor disorders |  |  |  |  |  |  | 1.9 | 0.01 |
| C3* | microglia-dependent synaptic plasticity | 2.24 | 0.001 | 2.34 | 0.01 |  |  | 3.78 | 0.001 |
| ENPP1* | maintenance of stem cell phenotype |  |  |  |  |  |  | 1.75 | 0.001 |
| GFAP* | neuroinflammation; major depression (MDD) and schizophrenia | 1.92 | 0.001 |  |  |  |  |  |  |
| MIR548H2** | microRNA, regulation of stability and translation of mRNA |  |  | -2.73 | 0.001 |  |  |  |  |
| NEXN* | neuroplasticity through cytoskeleton, signal transduction at the neurovascular unit |  |  |  |  |  |  | 1.84 | 0.01 |
| PARM1* | potential oncogene; prognostic biomarker for colorectal cancer |  |  | 1.95 | 0.001 |  |  | 1.71 | 0.001 |
| RAB3B* | long-term depression; short-term plasticity; normal reversal learning |  |  |  |  |  |  | 2.13 | 0.01 |
| RNU4-2* | RNA, U4 small nuclear 2 | 1.93 | 0.01 | 2.15 | 0.01 | 1.85 | 0.01 | 2.30 | 0.01 |
| RNU6ATAC* | cellular stress; aggressive neuroblastoma |  |  | -1.77 | 0.01 |  |  |  |  |
| RNU7-53P* | diabetes and related traits | -1.82 | 0.01 |  |  |  |  |  |  |
| RNU7-47P* | pre-mRNA intron splicing regulation |  |  |  |  |  |  | -1.70 | 0.01 |
| SNORD114-10* | neurodevelopmental disorders |  |  | -2.34 | 0.001 |  |  |  |  |
| SNORA13* | rRNA modification | 2.24 | 0.001 | 2.3 | 0.001 | 1..85 | 0.001 | 2.25 | 0.001 |
| SUSD1* | epigenetic signature |  |  |  |  |  |  | 2.42 | 0.001 |
| TRHR* | serotoninergic neurotransmission |  |  | -3.48 | 0.001 |  |  |  |  |

Abbreviations: *fc*, fold change; *Verified by RT-qPCR; **Verified by Stem-loop RT-qPCR

HC, hippocampus
